# Compliance of clinical microbiology laboratories with recommendations for the diagnosis of bloodstream infections: Data from a nationwide survey in Italy

**DOI:** 10.1002/mbo3.1002

**Published:** 2020-02-03

**Authors:** Fabio Arena, Marta Argentieri, Paola Bernaschi, Giacomo Fortina, Vesselina Kroumova, Patrizia Pecile, Mario Rassu, Teresa Spanu, Gian Maria Rossolini, Carla Fontana

**Affiliations:** ^1^ Department of Clinical and Experimental Medicine University of Foggia Foggia Italy; ^2^ IRCCS Don Carlo Gnocchi Foundation Florence Italy; ^3^ Microbiology Unit Children's Hospital Bambino Gesù Rome Italy; ^4^ Italian Work Group for Infections in Critically Ill Patient (GliPac‐AMCLI) Milan Italy; ^5^ Infection Control Unit University Hospital "Maggiore della Carità" Novara Italy; ^6^ Clinical Microbiology and Virology Unit Florence Careggi University Hospital Florence Italy; ^7^ Microbiology and Virology Lab AULS 8 Berica S. Bortolo Hospital Vicenza Italy; ^8^ Institute of Microbiology Università Cattolica del Sacro Cuore Fondazione Policlinico Universitario Agostino Gemelli Rome Italy; ^9^ Department of Experimental and Clinical Medicine University of Florence Florence Italy; ^10^ Department Experimental Medicine University of Rome Tor Vergata Rome Italy; ^11^ Microbiology and Virology Lab Tor Vergata University Hospital Rome Italy

**Keywords:** bacteraemia, blood cultures, laboratory workflow, quality, standardization

## Abstract

In 2014, the Italian Working Group for Infections in Critically Ill Patient of the Italian Association of Clinical Microbiologists updated the recommendations for the diagnostic workflow for bloodstream infections (BSI). Two years after publication, a nationwide survey was conducted to assess the compliance with the updated recommendations by clinical microbiology laboratories. A total of 168 microbiologists from 168 laboratories, serving 204 acute care hospitals and postacute care facilities, were interviewed during the period January–October 2016 using a questionnaire consisting of nineteen questions which assessed the level of adherence to various recommendations. The most critical issues were as follows: (a) The number of sets of blood cultures (BC) per 1,000 hospitalization days was acceptable in only 11% of laboratories; (b) the minority of laboratories (42%) was able to monitor whether BCs were over or under‐inoculated; (c) among the laboratories monitoring BC contamination (80%), the rate of contaminated samples was acceptable in only 12% of cases;(d) the Gram‐staining results were reported within 1 hr since BC positivity in less than 50% of laboratories. By contrast, most laboratories received vials within 2–4 hr from withdrawal (65%) and incubated vials as soon as they were received in the laboratory (95%). The study revealed that compliance with the recommendations is still partial. Further surveys will be needed to monitor the situation in the future.

## Graphical abstract

We present the result of national survey during which microbiologists from 204 Italian hospitals have been interviewed on the application of guidelines concerning the proper use of blood cultures. Although the study showed some positive aspects, a full compliance with the guidelines is still far away, and more surveys will be needed to monitor the situation in the future.

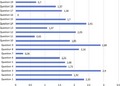

## 1 INTRODUCTION

The recent technological advances in diagnostic microbiology (e.g. the introduction of MALDI‐TOF‐based methods and of molecular biology‐based syndromic panels) have revolutionized the workflow of clinical microbiology laboratories (Brooks, 2013; Laupland & Valiquette, 2013; Opota, Corxatto, & Prod’hom G., Greub G., 2015). Concerning blood cultures (BCs), the average reporting time, can be significantly shortened providing clinicians with earlier information on infecting pathogens and their susceptibility profile that allow a more rapid revision or confirmation of the empirical therapy (Cohen et al., 2015; Liesenfeld, Lehman, Hunfeld, & Kost, 2014; Livermore & Wain, 2013; Maurer, Christner, Hentschke, & Rohde, 2017).

In 2014, the Italian Working Group for Infections in Critically Ill Patient of the Italian Association of Clinical Microbiologists (AMCLI) revised and updated the recommendations for the diagnostic workflow for bloodstream infections (BSIs), based on the most recent evidences (GLIPaC, 2014). The objectives of the revision included (a) reviewing the workflow in consideration of recent technological advances, (b) providing standard operating procedures (SOPs) for obtaining/processing BCs, and (c) identifying indicators that, assessed periodically, could be useful to monitor improvement in the diagnosis of BSIs. The document also underscored the importance of certain fundamental steps in both the pre‐analytical and analytical stages (De Plato et al., 2019; GLIPaC, 2014).

Two years after publication, we conducted a survey to evaluate the adherence to the recommendations by Italian clinical microbiology laboratories.

## 2 MATERIALS AND METHODS

### 2.1 Participants and data collection

A total of 168 microbiologists (from 168 laboratories) were interviewed. Altogether the laboratories served 204 hospitals and postacute care facilities (some laboratories acted as hubs for several hospitals). The data were collected from January to October 2016.

### 2.2 Survey

Each participant received a questionnaire with 19 questions and was given assistance in answering from the bioMérieux Italia Company specialists. All interviewed were bioMérieux customers who used the BACT/ALERT 3D BC monitoring system (bioMérieux). The questionnaires were collected and processed anonymously.

**Figure 1:**
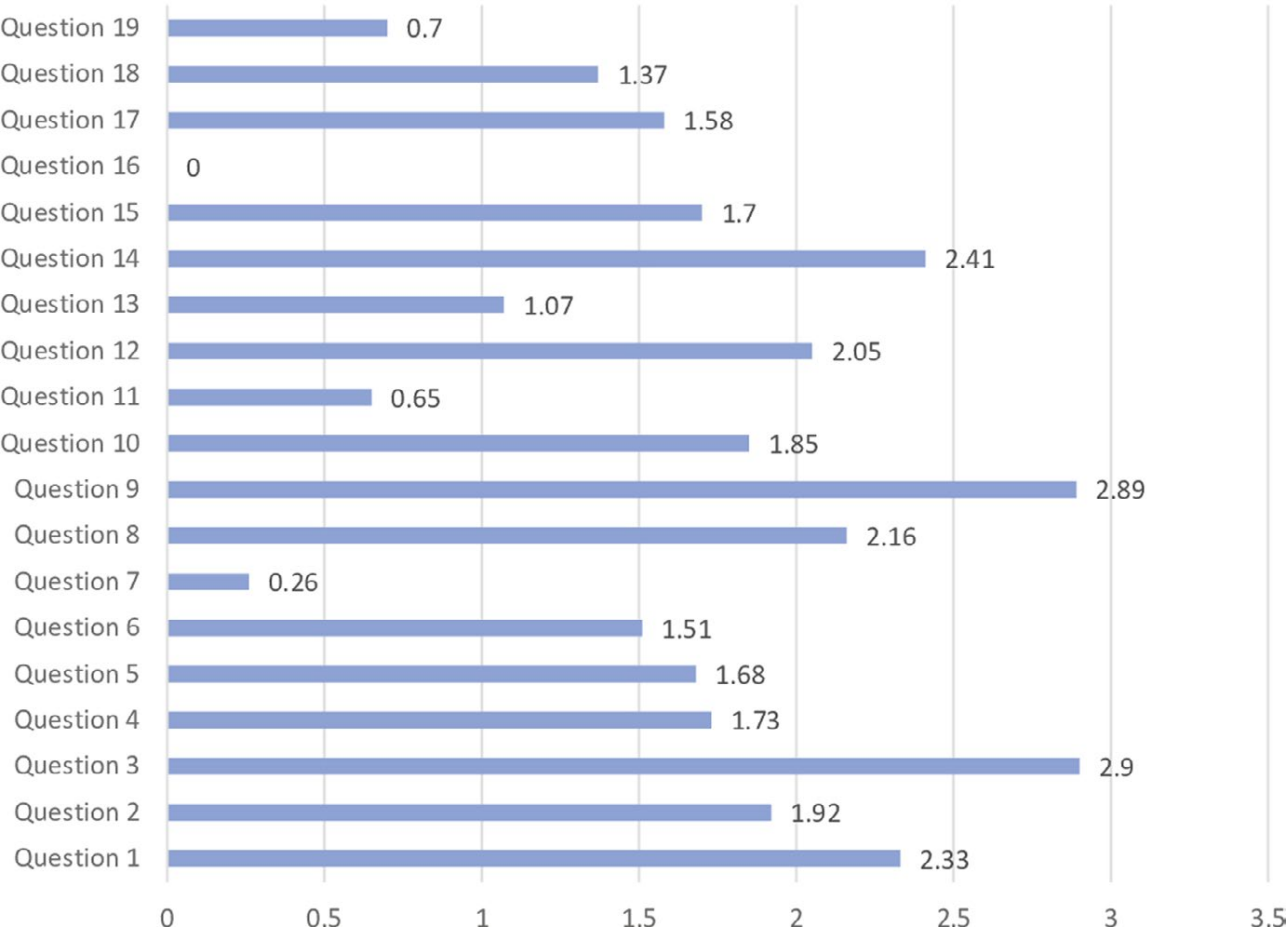
Average of the results for each question in all the centres interviewd. The value at the end of the bars indicates the average answered scored for each question (score ranging from 0 to 3, zero = no one answered)

Each question had four possible answers. For each choice, a score (ranging from 0 to 3) was assigned to grade the level of adherence of the assessed behavior to the updated recommendations (Table A1, Figure 1).

The questions were as follows:

1. How many samples are taken for each patient, with suspected bacteraemia, on the same day?
2. At what time distance from each other?
3. How many/which vials are inoculated for each sample?
4. What is the total volume of blood drawn for each patient on the same day?
5. Are repeated withdrawals performed for the same patient in days following the first?
6. What is the percentage of single blood cultures collected (in adult patients)?
7. How many blood culture sets are collected for 1,000 days of hospitalization?
8. What is the percentage of blood cultures delivered in the laboratory with a delay >2–4 hr from the time of sample collection?
9. What is the average time between the delivering of BCs to the laboratory and incubating them in the automatic systems?
10. What is the percentage of BCs obtained only from the central venous device and not accompanied by sampling from peripheral vein?
11. Can the percentage of overinoculated (>10 ml) or subinoculated (<8 ml) bottles be calculated? If so, report the prevalence?
12. What incubation duration has been set on the BC monitoring incubation system?
13. In case of suspected endocarditis or brucellosis, is the duration of the incubation prolonged?
14. Are positive bottles discharged from the instrument and managed as soon as possible or otherwise processed in batches at specific times of the day?
15. What is the average communication time for the Gram‐stain results (calculated from the moment a BC turned positive until the final reporting to the clinician)?
16. Do you adopt rapid identification methods and rapid antimicrobial susceptibility testing directly on positive broth culture? If so, which ones?
17. Does your laboratory information system record and manage (for statistical analysis) the positivity time for each bottle? If yes, reports the average.
18. What is your BC positivity rate?
19. What is your BC contamination rate? Do you produce cumulative reports as support?

Results were merged to calculate an average questionnaire score per center and an average answer score, for each question, intercenter.

## 3 RESULTS

Overall, 168 microbiologists from 168 laboratories were interviewed. The laboratories served a total of 204 acute care hospitals and postacute care facilities. The geographic distribution of the laboratories was as follows: 68 in northern Italy, 59 in central Italy, and 41 in southern Italy.

Figure 1 shows the average answer score for each question. Question no. 16 was excluded from the evaluation because of the low number of responses. Questions no. 7, 11, 13, and 19 yielded the lowest average scores. Among these, question no. 7 (no. of sets of blood cultures carried out for 1,000 days of hospitalization) yielded the lowest score. Only the 58% of laboratories (98/168) were able to monitor this parameter, and only in 11% (19/168), the parameter was in the expected range (Table 1). Answers to question no. 11 showed that a minority of laboratories (42%; 70/168) were able to monitor whether BCs were over or under‐inoculated, and among those able to perform monitoring, only a small number (14/70) had acceptable rates of inocula <2% (Table 1). Question no. 13, regarding the need to extend the incubation time in case of suspected brucellosis, showed heterogeneous behaviors, with 50% of laboratories reporting an extension of the incubation time, while the indication by the literature is to not extend the incubation time. *Brucella* spp., in fact, is able to grow within the traditional 5 days of incubation (GLIPaC, 2014; Habib, Lancellotti, & Iung, 2016; Lamy, Dargère, Arendrup, Parienti, & Tattevin, 2016). Concerning question no. 19, 89% of laboratories involved (150/168) were able to report data on contamination rate; only 12% (18/150) were in the expected range (2%–3%), while a significant number of hospitals were largely beyond scale (Table 1).

**Table 1:** Performances of the hospitals in monitoring some key indicators useful for verifying that the blood culture process is under control

Concerning question; (question no.)	Possible answer	No. of Hospital (%)^a^
Rate of blood cultures overinoculated (>10 ml) or subinoculated (<8 ml); (11)	>10%	7/70 (10)
	5%–10%	19/70 (27)
	2%–5%	30/70 (43)
	**<2%**	14/70 (20)
Timeline in reporting Gram‐stain results from positive blood cultures; (15)	>2 hr	20/168 (12)
	1−2 hr during the day and >2 hr in the night	25/168 (15)
	1−2 hr	24/168 (14)
	**<1 hr**	71/168 (42)
	Not reported	28/168 (17)
Blood culture positivity rate (% sets); (18)	<1% or > 19%	62/153 (41)
	1%–3% or 17%–19%	18/153 (12)
	3%–5% or 15%–17%	19/153 (12)
	**5%–15%**	54/153 (35)
Contamination rate of blood cultures (% sets); (19)^b^	>10%	51/150 (34)
	4%–10%	69/150 (46)
	3%–4%	12/150 (8)
	**≤2%–3%**	18/150 (12)

Note

In the column “Possible answer,” **bold** indicates the optimal answer.

a Referred to the number of centers that were able to answer.

b Evidences that may help to differentiate a contamination from a true bacteremia include: (a) identity of the microorganism (coagulase‐negative staphylococci [CoNS], *Corynebacterium* species, *Bacillus* species other than *anthracis*, *Propionibacterium acnes,* and *Micrococcus* species are usually considered contaminants); (b) number of positive culture sets; (c) number of positive bottles within a set; and (d) time to positivity.

**Figure 2:**
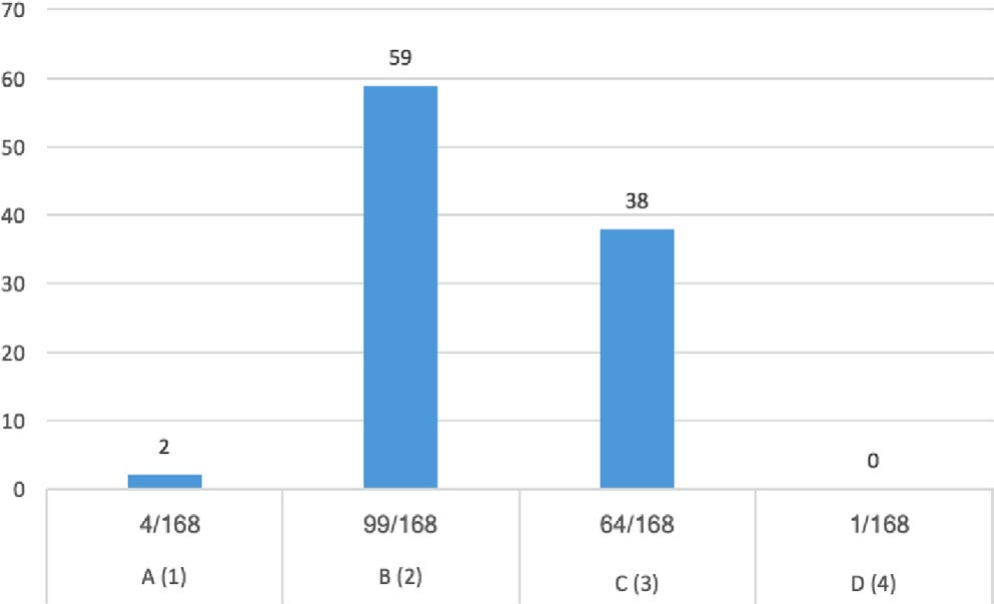
Number of set received for each BC, expressed in percentage for each Hospital. A (1) = one set; B (2) = two sets; C (3) = three sets; D (4) = four sets

**Figure 3:**
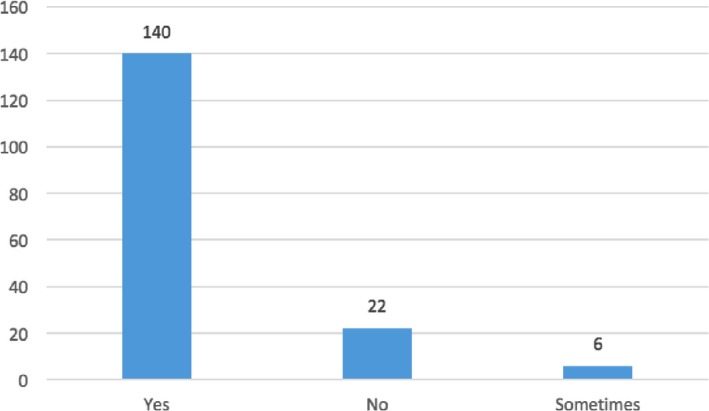
Hospital Adherence in Gram‐stain reporting. “yes” means that the microbiologist always reported Gram‐stain results; “no” that microbiologist never reported results; “sometimes” that microbiologist communicated results occasionally

Questions no. 1, 3, 8, 9, 12, and 14 were those which yielded an average answer score ≥2, indicating satisfactory adherence of the laboratories to the recommendations (Figure 1). In particular, question no. 1 assessed compliance with the best practice statement that strongly recommend the collection of at least two sets of BCs per patient (Lamy et al., 2016; Rhodes et al., 2017). These results were overall consistent with those yielded from question no. 3, about the number of vials inoculated for each sample (Figure 2). The high scores obtained for questions no. 8 and 9 suggest a good adherence to recommendations for the pre‐analytical phase in the BC workflow. In particular, question no. 8 revealed that the majority (110/168, 65%) of the laboratories received vials in an optimal time frame (between 2 and 4 hr). However, 40 laboratories were unable to evaluate this parameter, while the remaining 18 received vials with a delay exceeding 4 hr from the time of collection. Responses to question no.14 revealed that most laboratories (161 of 168, 95%) incubated BCs as soon as they were received in the laboratory. Finally, the answers to question no. 15 (time for Gram‐stain results reporting) showed an overall good compliance with the recommendation to perform and communicate Gram‐stain results on positive BCs as soon as possible (Clerc et al., 2013; GLIPaC, 2014; Thairu, Nasir, & Usman, 2014). Out of the 168 laboratories, 140 (83%) always reported Gram‐stain results, 28 (17%) either did not report at all or occasionally (Figure 3). More relevant, however, is the evaluation of the timing of communication of the results of microscopic observation (Table 1). In particular, 42% (71/168) of the laboratories reported the result of Gram‐stain from positive BCs in timely manner (score 3 was assigned to a reporting time ≤1 hr) and another 40% (20 + 25 + 24/168) reported the results with a significant delay (Table 1).

Answers to question no. 17 demonstrated that the majority of the laboratories calculated and reported the time to positivity (TTP) for each BC bottle, with 148/168 (88%) having these data available. This question included an assessment of the average TTP, which ranged from 15–30 hr for 108 laboratories, from 0 to 15 hr for five laboratories, and from 30 to 50 hr for 35 laboratories.

As for the positivity rate of BCs (question no. 18), most laboratories (91%; 153/168) monitored this parameter, but only 35% of them were within the expected positivity rate range (5%–15%) (Table 1).

## 4 DISCUSSION

Blood cultures remains the gold standard for the diagnosis of BSIs. As Miller et al. stated in their guidelines, "the diagnosis of bloodstream infections is one of the most critical functions of clinical microbiology laboratories" (Miller et al., 2018). Therefore, it is of fundamental importance for microbiologists, based on the available technological and human resources, to implement a diagnostic workflow capable of returning useful results to clinicians in the shortest possible timeframe to maximize impact on clinical decisions and patients outcomes (Serpa‐Pinto & Cardoso, 2014; Seymour et al., 2017; Yealy et al., 2015). Monitoring suitable indicators can contribute to these purposes (Lamy, Ferroni, Henning, Cattoen, & Laudat, 2018). Therefore, the rules and indicators reported in our recommendations should not be perceived as a burden for the laboratory but rather as a guidance to improve the use of BCs for the benefit of patients (GLIPaC, 2014).

With the intention to monitor the adherence to our document by the clinical microbiology laboratories and to identify areas for improvement, we conducted a fact‐finding survey in our country. From the data collected, several critical issues were detected, showing that adherence to the recommendations is still far from satisfactory. Some of these issues deserve a special attention. First, and probably the most important, is the deviation from the minimum required number of blood cultures ordered (the optimal is 103–108 per 1,000 hospital/days). This indicator, although not properly indicative of laboratory performance, can indicate correct/incorrect behaviors of clinicians in ordering BCs (EARSnet, 2012; Karch et al., 2015). A second critical issue is that a significant percentage of the laboratories perform the Gram‐stain and communicate the results in times longer than those recommended. It is well demonstrated from the literature, how this delay may impact on patient outcomes (Clerc et al., 2013; Thairu et al., 2014). A third critical issue concerns contamination rates, which deserves more awareness and attention. The optimal value is <3%, but only 10% of laboratories were in this range. Reducing the number of contaminated BCs avoids useless or even misleading reports (Bates, Goldman, & Lee, 1991; Dawson, 2014; Gander, 2009; Jakko, Hilt, & Bosboomb, 2013; Snyder et al., 2012). This parameter, which reflects the quality of withdrawal practices in terms of asepsis conditions during the collection of BCs, is also useful to understand when and where it is necessary to organize training courses for medical and/or nursing staff on methods for BC collection, storage, and transport standards (Rupp, Cavalieri, Marolf, & Lyden, 2017; Snyder et al., 2012). Another critical issue related with the performance of laboratories was that very few laboratories monitor the volume of blood inoculated in BCs.

In an era of remarkable technology innovation in clinical microbiology, a drastic reduction of reporting times is possible (Arena et al., 2016; Özenci & Rossolini, 2019). In this perspective, it is noteworthy that most laboratories were unable to answer question no. 16, which had the purpose of evaluating the diffusion of rapid diagnostic systems for BCs. Therefore, it could be useful to repeat this survey in the future, focusing on this aspect. Microbiologists should also be encouraged to better apply the SOPs on BCs before the next survey, to verify whether a call for adherence to the procedures is actually effective in achieving greater compliance.

## 5 CONCLUSIONS

In conclusion, optimal practices of BC sampling and processing require thorough understanding of several issues. Quality control programs, including software‐based controls of pre‐ and postanalytical variables, should be strengthened to address the shortcomings described by numerous authors and also emerged in our study. We hope that the results of this first survey could encourage microbiologists to improve adherence to BC guidelines and recommendations.

## CONFLICT OF INTEREST

Carla Fontana has received a research grant by Quintiles/Angelini. Advisory Board: Angelini, Pfizer. Fabio Arena has received congress lecture fees from Accelerate Diagnostics, Alifax, Angelini ACRAF, Astellas, bioMérieux, Cepheid. Gian Maria Rossolini has received research grants from Accelerate Diagnostics, Alifax, Angelini ACRAF, AstraZeneca, Basilea, Becton‐Dickinson, bioMérieux, Biotest, Cepheid, Checkpoints, Elitech, Liofilchem, Merck, Novartis, Nordic Pharma, Pfizer, Rempex/The Medicine Company, Zambon; has received congress lecture fees from Angelini ACRAF, AstraZeneca, Basilea, Biotest, Merck, Pfizer; has received consultancy fees from Achaogen, Angelini ACRAF, AstraZeneca, Curetis, Elitech, Menarini, Merck, Nordic Pharma, Pfizer, Rempex/The Medicine Company, Zambon. The other authors have no other relevant affiliations or financial involvement with any organization or entity with a financial interest in or financial conflict with the subject matter or materials discussed in the manuscript apart from those disclosed.

## AUTHOR CONTRIBUTIONS

Fabio Arena: Conceptualization‐Equal, Data curation‐Equal, Formal analysis‐Equal, Investigation‐Equal, Methodology‐Equal, Project administration‐Lead, Supervision‐Lead, Writing‐original draft‐Equal, Writing‐review & editing‐Lead; Marta Argentieri: Data curation‐Equal, Formal analysis‐Equal, Investigation‐Supporting; Paola Bernaschi: Data curation‐Equal, Formal analysis‐Equal, Investigation‐Equal; Giacomo Fortina: Data curation‐Equal, Formal analysis‐Equal, Investigation‐Equal; Vesselina Kroumova: Data curation‐Equal, Formal analysis‐Equal; Patrizia Pecile: Data curation‐Supporting; Mario Rassu: Data curation‐Supporting, Investigation‐Equal; Teresa Spanu: Data curation‐Equal, Investigation‐Equal; Gian Maria Rossolini: Conceptualization‐Equal, Formal analysis‐Equal, Investigation‐Equal, Supervision‐Lead; Carla Fontana: Conceptualization‐Equal, Data curation‐Equal, Formal analysis‐Equal, Investigation‐Equal, Methodology‐Equal, Project administration‐Equal, Supervision‐Lead, Validation‐Equal, Visualization‐Equal, Writing‐original draft‐Equal, Writing‐review & editing‐Lead.

## ETHICS STATEMENT

A written informed consent to publish the data was obtained from all survey participants.

## Data Availability

All data are provided in full in the results section of this paper.

## APPENDIX 1

**Table A1:** Scores assigned to each possible response are based on adherence of the assessed behavior to the updated recommendations

	Score
Question 1: How many samples are taken for each patient on the same day?	
Answers	
1 Sample	0
2 Samples	2
3 Samples	3
4 Samples	1
Question 2: At what temporal distance from each other?	
Answers	
Withdrawals spaced from >60 min and after empirical therapy and regardless of when antibiotic therapy is given	0
Withdrawals spaced from 30–60 min and after empirical therapy and regardless of when antibiotic therapy is given	1
Withdrawals taken at a distance ≤30–60 min before the start of empirical therapy or in any case before a new administration	2
Close sampling (5–10 min) before the start of empirical therapy or in any case before a new administration	3
Question 3: How many/which vials are inoculated for each sample?	
Answers	
1 Single adult‐bottle for both adults and children	0
1 Single adult‐bottle and one dedicated bottle for pediatrics	1
2 Bottles for adults and one adult bottle also used for pediatric sampling	2
2 Bottles for adults and one bottle for children	3
Question 4: What is the total volume of blood taken for each patient on the same day?	
Answers	
<5 ml	0
>40 ml and < 20 ml	1
30–40 ml	2
20–30 ml	3
Question 5: Are repeated withdrawals performed for the same patient in days following the first?	
Answers	
No, never	0
Yes, often/always even in the absence of clinical data	1
Yes, but only in the presence of relevant clinical data	2
Yes, but only in some cases as sepsis from S. aureus, to guide therapy in case of candidemia, endocarditis in case of negativity of the first three sets or in the presence of relevant clinical data	3
Question 6: What is the percentage of single blood cultures collected (in adult patients)?	
Answers	
>10%	0
5%–10%	1
3%–5%	2
0%–3%	3
Question 7: How many blood‐culture sets are collected for 1,000 days of hospitalization?	
0–50 and >250	0
220–250	1
50–103 and 188–220	2
Ranging between 103 and 188	3
Question 8: What is the percentage of blood cultures delivered in the laboratory with a delay >2–4 hr from the time of the sample collection?	
not defined	
Question 9: What is the average time between the delivery of BCs in the laboratory and its incubation into the automatic systems?	
Answers	
>4 hr	0
3–4 hr	1
2–3 hr	2
<2 hr	3
Question 10: What is the percentage of BCs taken only by central venous device and not accompanied by peripheral vein withdrawal?	
Answers	
>7%	0
5%–7%	1
About 5%	2
<5%	3
Question 11: Can you calculate the percentage of over‐inoculated (>10 ml) or sub‐inoculated (<8 ml) bottles? If so, what is its prevalence?	
Answers	
>10%	0
5%–10%	1
2%–5%	2
<2%	3
Question 12: What incubation duration has been set on your BC monitoring incubation system?	
<5 and >7 days	0
7 days	1
6 days	2
5 days	3
Question 13: In case of suspected endocarditis or brucellosis, is the duration of the incubation prolonged?	
Yes	0
No	3
Question 14: Are positive bottles downloaded from the instrument and managed as soon as possible or otherwise they are processed in batch at specific times of the day?	
Batch removal of positive bottles	0
Positive‐bottles are discharged every 1–2 hr during the day and >2 hr at night	1
Positive‐bottles are removed every 1−2 hr	2
Positive bottles are removed as soon are flagged positive	3
Question 15: What is the average time of communication of the Gram‐stain results (calculated starting from the time a BC turned positive to the final reporting to the clinician)?	
>2 hr	0
1−2 hr along the day and >2 hr in the night	1
1−2 hr	2
<1 hr	3
Question 16: Do you adopt rapid identification methods and rapid antimicrobial susceptibility testing directly on positive broth culture? If so, which ones?	
Not valuable	
Question 17: Does your laboratory information system record and manage (for statistical analysis) the times to positivity for each bottle? If yes, reports the average.	
>50 hr	0
30−50 hr	1
15−30 hr	2
0−15 hr	3
Question 18: What is your BC positive rate?	
<1% or >19%	0
1%–3% or 17%–19%	1
3%–5% or 15%–17%	2
5%–15%	3
Question 19: What is your BC contamination rate? Do you produce cumulative reports as support?	
>10%	0
4%–10%	1
3%–4%	2
≤2%–3%	3
